# Nanocarrier-Based Ocular Drug Delivery Systems for Retinal Diseases: Therapeutic Potential

**DOI:** 10.3390/life16050810

**Published:** 2026-05-13

**Authors:** Dominika Skarbek, Alicja Sochocka, Oliwia Sidło, Aleksandra Sapiaszko, Agnieszka Drab, Jacek Baj, Robert Rejdak, Joanna Dolar-Szczasny

**Affiliations:** 1 Student Scientific Club at the Department of General and Paediatric Ophthalmology, Medical University of Lublin, Chmielna 1, 20-079 Lublin, Poland; 63367@umlub.edu.pl (A.S.); 63295@umlub.edu.pl (O.S.); 63290@umlub.edu.pl (A.S.); 2 Department of Medical Informatics and Statistics with e-Health Lab, Medical University of Lublin, Jaczewskiego 4, 20-090 Lublin, Poland; agnieszka.drab@umlub.edu.pl; 3 Department of Correct, Clinical and Imaging Anatomy, Medical University of Lublin, Jaczewskiego 4, 20-090 Lublin, Poland; jacek.baj@umlub.edu.pl; 4 Department of General and Paediatric Ophthalmology, Medical University of Lublin, Chmielna 1., 20-079 Lublin, Poland; robert.rejdak@umlub.edu.pl

**Keywords:** ocular drug delivery, nanocarriers, retinal diseases, intravitreal delivery, liposomes, polymeric nanoparticles, PLGA, targeted drug delivery

## Abstract

Background: Posterior segment eye diseases, including age-related macular degeneration and diabetic retinopathy, are preeminent causes of vision loss worldwide. Effective drug delivery to the retina poses an ongoing therapeutic difficulty due to the presence of the anatomical and physiological barriers. Nanotechnology-based drug delivery systems represent a promising strategy to overcome those limitations. Methods: A narrative literature review was conducted using the PubMed, Scopus, and Google Scholar databases, covering publications published between 2019 and 2026. Publications evaluating nanoparticles for the treatment of the vitreoretinal disorders, including pre-clinical in vitro and in vivo studies, were analyzed. Results: Nanocarriers, including liposomes, polymeric nanoparticles, and lipid-based systems, established improved drug bioavailability, stability, and targeted delivery. The analyzed systems facilitate sustained drug release and potentially reduce the prevalence of invasive intravitreal injections. The nanocarriers’ effectiveness is primarily influenced by their physicochemical properties, such as particle size, surface charge, and encapsulation efficiency. Nonetheless, the production costs and safety aspects, including cytotoxicity, oxidative stress, and inflammatory responses, remain as significant limitations. Conclusions: Nanotechnology-based drug delivery systems serve as an auspicious therapeutic approach for posterior segment eye diseases. However, further standardized preclinical and clinical research is required to assure long-term safety and enable successful clinical transition.

## 1. Introduction

Retinal diseases are among the leading causes of vision loss worldwide. The most significant conditions in this group include age-related macular degeneration (AMD) and diabetic retinopathy (DR). In 2020, AMD was estimated to affect approximately 196 million people, while DR affected about 146 million [1]. The increase in the prevalence of these diseases is closely linked to the aging population and the growing global number of people with diabetes. Due to their high prevalence and serious impact on patients’ quality of life, both diabetic retinopathy and AMD remain the focus of intensive research into their pathogenesis, early diagnosis, and new treatment methods [2,3].

Standard treatment for eye diseases primarily involves the topical application of medication in the form of eye drops. This method is minimally invasive and readily available; however, due to the eye’s unique protective barriers, it does not yield sufficient results [4,5]. In the treatment of retinal diseases, invasive drug delivery methods such as intravitreal injections (IVT) and implants have therefore begun to be introduced. A significant drawback of these methods is the risk of potential complications, such as inflammation, intraocular hemorrhage, or mechanical damage to ocular structures. Implants provide prolonged drug release, which reduces the frequency of drug administration. If patients experience side effects, the implants must be surgically removed, which may increase healthcare costs. IVT, on the other hand, requires monthly or bimonthly injections. Consequently, researchers highlight the emerging need for new therapies and techniques for delivering drugs to the eye to improve the quality of patient care [6,7,8].

**Figure 1:**
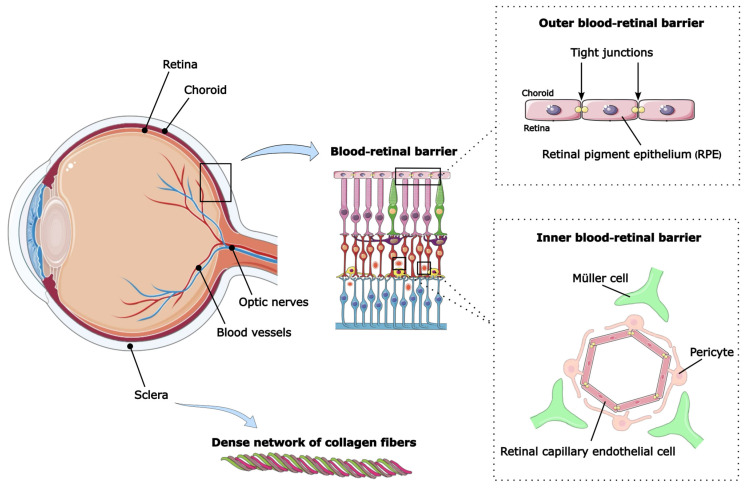
Schematic diagram of the posterior ocular tissues barriers of the eye. (This figure was partly generated using Servier Medical Art, provided by Servier (Suresnes, France), licensed under a Creative Commons Attribution 4.0 license).

Although the eye is anatomically accessible, drug delivery remains challenging. The human eye can be divided into two segments, each with its own specific physiological and anatomical protective barriers (Figure 1). The anterior segment consists of the cornea, conjunctiva, iris, ciliary body, lens, and aqueous humor. The posterior ocular tissues, on the other hand, contain the sclera, choroid, retina, vitreous humor, and optic nerve [3,9]. In the posterior segment of the eye, the barrier formed by the sclera is significant in terms of drug bioavailability. The sclera consists of a dense network of collagen fibers and proteoglycans, limiting the penetration of substances into the eye. Its thickness varies regionally, measuring approximately 0.53 mm at the corneoscleral limbus, decreasing to approximately 0.39 mm near the equator, and increasing again to 0.9–1.0 mm in the peripapillary region near the optic nerve [9]. Additionally, this layer has a negative electrostatic charge, which may hinder the penetration of positively charged compounds [10]. The vitreous humor, which fills the space between the lens and the retina, presents a further diffusional barrier: the mean vitreous chamber depth in adults is approximately 15.7 mm [11], a cavity that nanocarriers must traverse to reach retinal targets. Another major limitation to drug penetration is the blood–retinal barrier (BRB), which separates the systemic circulation from retinal tissue. It is divided into an inner (iBRB) and outer (oBRB) blood–retinal barriers. The oBRB consists of the choroid, Bruch’s membrane, and the retinal pigment epithelium (RPE) [12]. Bruch’s membrane is a five-layered modified connective tissue approximately 2–4 µm thick, forming a molecular sieve that regulates the exchange of nutrients and metabolites between the choriocapillaris and the RPE [13]. The RPE, which forms the outermost cellular layer of the retina, consists of a single monolayer of hexagonal cells; in the central macula, RPE cells measure approximately 12–18 µm in height and ~10 µm in width, becoming broader and flatter toward the periphery [14]. The inner BRB, on the other hand, consists of retinal capillary endothelial cells supported by Müller endfoot processes [15]. Key components of the blood–retinal barrier are the so-called tight junctions (TJ), which form specialized intercellular connections between the retinal capillary endothelium that form a selective barrier limiting diffusion and transport into the intraocular space [12,16]. The choroid, which plays a role in nourishing the outer retina, determines the rapid elimination of drugs. These barriers significantly hinder the development of effective therapeutic strategies [3,10].

Nanoparticle-based delivery systems represent a promising therapeutic strategy. They enable higher drug concentrations and greater stability, as well as improved penetration into specific eye tissues, while reducing the risk of adverse effects [5]. Nanocarriers used in ophthalmology include: poly(lactic-co-glycolic) acid (PLGA), nanomicelles, lipid nanoparticles, liposomes, hydrogels, and microneedle-based systems [3,17]. To adequately assess the effectiveness of these approaches, it is necessary to objectively compile the results from the literature, with particular emphasis on the preclinical phase. These studies, conducted under laboratory conditions and in animal models, provide insight into the drug’s mechanisms of action, its pharmacokinetics, and potential adverse effects. These studies form the basis for safety assessment prior to the initiation of clinical trials [18].

This narrative review focuses on an analysis of preclinical scientific evidence regarding the use of nanotechnology in drug delivery systems targeting the retinal tissues of the eye. A critical evaluation of the available literature aims to verify the efficacy and application potential of specific nanotechnological solutions.

## 2. Materials and Methods

A literature search was conducted using PubMed, Scopus, and Google Scholar databases. The following keywords and combinations were applied: “nanoparticles”, “nanocarriers”, “nanomedicine”, “ocular drug delivery”, “retinal diseases”, “posterior segment”, “intravitreal delivery”, “liposomes”, “polymeric nanoparticles”, and “PLGA”. Boolean operators (AND, OR) were used to refine the search strategy. The primary search included articles published between 2019 and 2026; however, earlier seminal studies were also included to provide essential background context. This review included scientific articles describing the use of nanotechnology in the delivery of therapeutic agents to the posterior segment of the eye, particularly in the context of retinal diseases. Both review articles and original research articles on in vitro, in vivo, and preclinical studies were included. The inclusion criteria were publications available in English, with full-text access. Reference lists of relevant articles were also screened to identify additional studies.

Publications not related to drug delivery to the retinal tissues, popular science articles, conference abstracts, letters to the editor, and duplicates were excluded from the analysis. The selected publications were subjected to qualitative analysis, focusing on the types of nanocarriers used, mechanisms enhancing drug bioavailability, and their potential therapeutic applications in retinal diseases.

The aim of this review is to provide a concise overview of the most important research areas and current trends in the development of nanocarriers for the treatment of vitreoretinal diseases. Ultimately, a total of 30 articles were included in the review. Of these, 20 articles are discussed in the Results Section with specific findings reported; the remaining 10 articles inform the Discussion Section with supporting evidence on physicochemical determinants, safety profiles, and translational considerations.

## 3. Results

### 3.1. Liposomes

Liposomes represent a widely studied class of nanocarrier systems for ocular drug delivery, typically ranging from 80 to 1000 nm in diameter [19]. Structurally, they consist of one or more phospholipid bilayers surrounding an aqueous core. Their composition, which includes phospholipids and cholesterol, resembles biological membranes. Preclinical studies have reported favorable biocompatibility and low toxicity in ocular tissues [19,20]. Due to their amphiphilic structure, liposomes can encapsulate both hydrophilic and hydrophobic therapeutic agents, enabling protection of the drug from degradation and modulation of its pharmacokinetic profile. Positively charged liposomal formulations have been shown to exhibit increased interaction with negatively charged ocular surfaces, resulting in prolonged residence time and improved permeability [19]. However, limitations such as rapid systemic clearance, off-target accumulation, and potential immunogenicity have also been reported [20].

In the context of diabetic retinopathy, liposome-based systems have been evaluated in preclinical models. In a study by Amato et al. (2023) [21], liposome-encapsulated Litosan G (LipoLG) was administered in a streptozotocin-induced diabetic mouse model. The results demonstrated reduced levels of active caspase-3 and prevention of VEGF upregulation compared to non-encapsulated formulations, indicating enhanced bioavailability of the active compound [21].

### 3.2. Polymeric Nanoparticles (PLGA)

Polymeric nanocarrier systems, particularly those based on PLGA, are frequently used in ocular drug delivery due to their biodegradability and controlled release properties. The physicochemical properties of PLGA systems can be modified by adjusting the lactic-to-glycolic acid ratio. These systems undergo hydrolysis into lactic and glycolic acids, which are metabolized via physiological pathways. Preclinical studies have demonstrated low cytotoxicity and the ability to cross ocular barriers [22,23,24]. However, limited mucoadhesive properties have been reported, particularly in topical administration [24].

In a study by Liu et al. (2020) [25], a PLGA-based nanocarrier system incorporating dexamethasone and bevacizumab was developed and functionalized with a cRGD peptide. The nanoparticles exhibited an average size of approximately 213 nm. In vitro experiments using ARPE-19 cells demonstrated inhibition of angiogenesis and cellular migration, as well as induction of apoptosis in pathological cells [25].

PLGA-based systems have also been evaluated in diabetic retinopathy models. In a study by Qiu et al. (2019) [26], fenofibrate-loaded PLGA nanocarriers were administered via a single intravitreal injection in diabetic rats. After eight weeks, improved retinal function, reduced vascular leakage, decreased leukostasis, and downregulation of VEGF and ICAM-1 expression were observed. Sustained drug release was also reported, indicating prolonged therapeutic activity [26].

### 3.3. Targeted Nanocarriers

Targeted nanocarrier systems have been developed to improve selective delivery to retinal tissues. Surface functionalization with ligands such as peptides or antibodies enables receptor-mediated uptake and enhances cellular internalization. For example, cRGD-functionalized PLGA nanocarriers have been shown to exhibit increased affinity for αVβ3 integrin receptors expressed on retinal pigment epithelial cells under pathological conditions [25].

These systems demonstrated improved targeting efficacy, inhibition of angiogenesis, and reduced cellular migration in in vitro models. Functionalization strategies, including ligand conjugation and surface modification, have also been associated with enhanced penetration across ocular barriers and improved localization within retinal tissues [25].

### 3.4. Lipid Nanoparticles and Solid Lipid Nanoparticles

Lipid nanoparticles (LNPs), including solid lipid nanoparticles (SLNs) and nanostructured lipid carriers (NLCs), represent a versatile class of ocular nanocarriers that combine the biocompatibility of physiological lipids with the ability to encapsulate both hydrophilic and lipophilic drugs. SLNs are composed of a solid lipid matrix stabilized by surfactants, typically ranging from 50 to 400 nm in diameter [3]. Their solid lipid core provides sustained drug release and protects labile molecules from degradation. NLCs, a second-generation development, incorporate a mixture of solid and liquid lipids, resulting in imperfect crystal matrices with higher drug-loading capacity and improved stability compared to SLNs [10]. Preclinical studies have demonstrated favorable biocompatibility of lipid nanoparticles in ocular tissues, with several formulations showing enhanced drug penetration into the posterior segment following topical or intravitreal administration. For example, LNPs loaded with antioxidant compounds such as lutein and coenzyme Q10 have been investigated for neuroprotective applications in retinal degeneration models, demonstrating improved retinal bioavailability compared to free drug solutions [3,5].

### 3.5. Nanomicelles

Polymeric nanomicelles are self-assembling nanostructures formed by amphiphilic block copolymers, typically 10–100 nm in diameter, which spontaneously organize into core–shell architectures in aqueous environments. Their small size facilitates deep tissue penetration and diffusion through the vitreous humor. The hydrophobic core enables solubilization of poorly water-soluble ophthalmic drugs such as steroids (e.g., triamcinolone acetonide, dexamethasone) and anti-VEGF small molecules, while the hydrophilic shell promotes prolonged ocular surface residence time and reduced non-specific cellular uptake [27]. Preclinical studies using polymeric nanomicelles incorporating tacrolimus and cyclosporine A have demonstrated enhanced corneal and scleral permeation compared to conventional eye drop formulations. In the context of retinal diseases, nanomicelles have been reported to improve bioavailability of antioxidant compounds, including vitamin E and N-acetylcysteine, in both in vitro and in vivo models of oxidative stress-induced retinal damage [27,28].

### 3.6. Metal-Based Nanoparticles

Metal-based nanoparticles, including gold nanoparticles (AuNPs), silver nanoparticles (AgNPs), and cerium oxide nanoparticles (nanoceria), have been explored for ocular drug delivery and theranostic applications. Gold nanoparticles are of particular interest due to their tunable surface chemistry, enabling conjugation of targeting ligands, nucleic acids, or therapeutic peptides, as well as their photothermal properties that are applicable to laser-assisted drug release strategies. AuNPs in the range of 10–50 nm have demonstrated the ability to penetrate the BRB and accumulate in retinal tissues following intravitreal administration [5]. Cerium oxide nanoparticles possess intrinsic antioxidant properties owing to their redox-active Ce^3+^/Ce^4+^ valence states, making them attractive candidates for neuroprotective applications in AMD and DR models, where oxidative stress is a key pathological driver. However, safety concerns remain regarding the long-term cytotoxicity and inflammatory potential of metal-based nanoparticles, as evidenced by studies reporting silver nanoparticle-induced apoptosis in ARPE-19 retinal cells at elevated concentrations [29,30]. Careful size and surface charge optimization, as well as surface coating with biocompatible polymers, are critical to improving their safety profiles for ocular use.

### 3.7. Hydrogel-Based and Microneedle-Based Systems

Hydrogel-based nanocarrier systems represent an emerging approach for sustained ocular drug delivery, combining the properties of polymeric networks with controlled drug release kinetics. Stimuli-responsive hydrogels (e.g., temperature-, pH-, or light-responsive systems) enable on-demand drug release in response to the local ocular microenvironment. Nanoparticles embedded within hydrogel matrices can further prolong drug release and improve retention at the administration site. For instance, nanoparticle-loaded hyaluronic acid hydrogels have demonstrated sustained anti-VEGF agent release in preclinical AMD models, reducing the frequency of intravitreal injections required for therapeutic effect [31,32]. Microneedle-based delivery systems offer a minimally invasive route for posterior segment drug delivery, enabling transscleral or suprachoroidal access to retinal tissues. Hollow or dissolving microneedle arrays can be loaded with drug-encapsulating nanoparticles, allowing precise spatial delivery while avoiding the risks associated with conventional intravitreal injections. Recent studies have described microneedle-mediated delivery of biomimetic nanoparticles loaded with antioxidant and anti-inflammatory agents to retinal tissue in AMD models, demonstrating improved therapeutic outcomes compared to free drug controls [33].

### 3.8. Comparative Efficacy and Physicochemical Determinants

The therapeutic performance of nanocarrier systems is influenced by their physicochemical properties, including particle size, polydispersity index (PDI), surface charge, and encapsulation efficiency (EE%) (key terms defined in Table 1). Studies have shown that particles in the range of 50–400 nm demonstrate favorable penetration and stability profiles [34,35]. Smaller particles (<200 nm) have been associated with improved tissue penetration, whereas larger particles (>300 nm) may exhibit increased aggregation [36]. Additionally, particle size influences distribution within ocular tissues, with smaller particles demonstrating greater penetration into retinal layers [28].

These size–function relationships were established through a combination of ex vivo vitreous diffusion assays, fluorescence microscopy studies in enucleated eyes, and in vivo distribution studies in rodent and rabbit models. Diffusion through the vitreous humor is particularly sensitive to particle size: nanoparticles below 200 nm move relatively freely through the vitreous gel network, whereas particles above 500 nm undergo significant entrapment within the collagen–hyaluronic acid matrix [28,32,36]. The size ranges reported in the studies included in this review are consistent with these established constraints; however, it should be noted that optimal size may vary depending on the administration route (e.g., intravitreal vs. suprachoroidal vs. topical) and the specific retinal target tissue.

PDI reflects the uniformity of particle size distribution. Values below 0.2 have been associated with homogeneous systems and improved stability, whereas higher values may indicate increased aggregation risk [27,37]. In a study by Beirampour et al., PLGA nanocarriers with a PDI below 0.2 and a size range of 98–288 nm maintained physicochemical stability despite a negative zeta potential [37].

EE% is another critical parameter affecting drug delivery. High EE% values have been associated with prolonged drug release and improved therapeutic outcomes. For example, PLGA-based systems containing anti-VEGF agents demonstrated sustained release profiles compared to free drug formulations [33]. Similarly, liposomal formulations have shown increased drug concentrations in retinal tissues compared to conventional ophthalmic solutions [38]. The key physicochemical and efficacy parameters of the nanocarrier systems reviewed herein are summarized in Table 2.

Although nanocarrier systems have demonstrated favorable biocompatibility in preclinical models, studies have also reported potential adverse effects, including oxidative stress, inflammation, and cytotoxicity, depending on nanoparticle composition and physicochemical characteristics [5,28,29,30,39]. These findings highlight the importance of optimization of formulation parameters and conducting thorough safety evaluation.

## 4. Discussion

### 4.1. Therapeutic Potential of Nanocarrier-Based Systems in Retinal Diseases

Nanocarrier-based systems are increasingly investigated as advanced strategies for the treatment of retinal diseases, including age-related macular degeneration and diabetic retinopathy. The available evidence indicates that these delivery platforms can enhance drug penetration across ocular barriers and enable sustained release, potentially reducing the frequency of intravitreal administration. In addition, nanocarriers facilitate targeted delivery to retinal tissues and may improve local drug bioavailability compared to conventional formulations [38,40]. For example, in a preclinical study by Amato et al., liposome-encapsulated Litosan G demonstrated improved bioavailability and reduced apoptotic markers compared to non-encapsulated formulations [21]. Similarly, Liu et al. reported that PLGA-based nanocarriers functionalized with cRGD effectively inhibited angiogenesis and cellular migration in retinal cell models [25].

### 4.2. Current Approaches to Retinal Drug Administration

Intravitreal injection remains the most effective route for delivering therapeutic agents to the retina, as it allows direct access to target tissues while bypassing systemic clearance mechanisms [28,41,42]. However, this approach is invasive and associated with complications such as retinal detachment, endophthalmitis, vitreous hemorrhage, and reduced patient compliance due to repeated procedures [19,28,33,42]. The cumulative burden of repeated injections is substantial: the reported incidence of endophthalmitis following intravitreal injection ranges from approximately 0.019% to 0.077% per injection, and the risk of rhegmatogenous retinal detachment, vitreous hemorrhage, cataract progression, and elevated intraocular pressure increases with injection frequency [19,42]. Beyond procedural complications, repeated injections impose a significant psychological burden on patients, contribute to reduced treatment adherence, and generate considerable healthcare costs. Intraocular implants, while reducing administration frequency, require surgical placement and, in the event of adverse reactions, surgical removal—further adding to the procedural risk and cost burden [6,7]. In contrast, nanocarrier-based delivery systems offer several favorable properties that directly address these limitations: they enable sustained, controlled drug release over weeks to months from a single administration, thereby reducing the required injection frequency; they can be engineered to improve drug targeting and minimize systemic exposure; and their submicron dimensions may facilitate less traumatic administration routes, including suprachoroidal or periocular delivery [5,33,40]. It is important to note that intravitreal injection itself is not eliminated as an administration route in nanocarrier-based approaches—rather, the primary goal is to reduce the frequency of injections required to maintain therapeutic drug concentrations within the target tissue. Alternative strategies, including sustained-release implants, microneedle-based delivery, and iontophoresis, have been investigated to improve safety and reduce treatment burden. These approaches may enable less invasive administration while maintaining therapeutic drug concentrations in retinal tissues [31,33,40,41,43]. This is supported by the study of Qiu et al., in which intravitreal administration of fenofibrate-loaded PLGA nanocarriers resulted in sustained therapeutic effects for up to eight weeks, along with reduced vascular leakage and inflammatory markers [26].

### 4.3. Biological Barriers and Mechanisms of Retinal Drug Transport

Effective drug delivery to retinal tissues requires overcoming multiple biological barriers, including the vitreous humor and the blood–retinal barrier. The physicochemical properties of nanocarriers play a critical role in this process. Surface charge influences mobility within the vitreous, where negatively charged or neutral systems demonstrate improved diffusion compared to cationic carriers, which may undergo aggregation [32,44]. In addition, transport across retinal tissues occurs via paracellular and transcellular pathways, with receptor-mediated mechanisms enhancing cellular uptake. Functionalization strategies, such as ligand conjugation or surface modification with targeting moieties, have been shown to improve delivery efficiency [32,45,46,47]. Experimental evidence also indicates that nanoparticle size significantly influences retinal penetration, with smaller carriers (<200 nm) demonstrating improved distribution within retinal layers [28].

### 4.4. Formulation Parameters Influencing Nanocarrier Performance and Safety Aspects

Particle size and formulation homogeneity are key determinants of nanocarrier performance. Smaller particles generally demonstrate improved tissue penetration, whereas larger particles may exhibit prolonged retention but increased aggregation risk [28,34,35,36,38]. Similarly, a low polydispersity index is associated with greater formulation stability and more predictable biodistribution [27,37]. Encapsulation efficiency also plays an important role, as higher drug loading enables sustained release and prolonged therapeutic activity [19,33,37,38]. These parameters require careful optimization to achieve an optimal balance between efficacy, stability, and safety.

Despite promising preclinical outcomes, several challenges remain. The majority of available data are derived from in vitro and animal studies, and their translation to clinical practice remains limited. In addition, nanocarrier systems may induce adverse effects, including oxidative stress, inflammation, and cytotoxicity, depending on their composition and surface characteristics. Several studies have reported such effects, highlighting the need for careful safety evaluation and standardization of nanoparticle formulations [5,29,30,39]. Manufacturing complexity and high production costs further limit large-scale implementation [38,40].

### 4.5. Future Perspectives

Overall, nanocarrier-based ocular drug delivery systems represent a promising approach for improving the treatment of retinal diseases. However, further research is required to standardize formulations, validate long-term safety, and facilitate clinical translation.

Future research should focus on the translation of nanocarrier-based systems from preclinical models to clinical practice, with particular emphasis on safety, long-term toxicity, and large-scale manufacturing [48]. The integration of targeted delivery strategies and stimuli-responsive nanocarriers may further enhance therapeutic efficacy and reduce systemic exposure. In addition, advances in personalized medicine and biomarker-driven approaches may enable the development of more individualized and effective ocular drug delivery systems.

### 4.6. Limitations

This review has several limitations. First, the included studies are heterogeneous in terms of experimental design, nanoparticle types, and outcome measures, which limits direct comparison between studies. Second, most of the available evidence is based on in vitro and animal models, and therefore may not fully reflect human ocular physiology. Third, the lack of standardized protocols for nanoparticle characterization and evaluation complicates the interpretation of results. Finally, potential publication bias and the limited number of clinical studies should also be considered when interpreting the findings.

Despite these limitations, the available evidence suggests that nanocarrier-based systems represent a promising strategy for improving ocular drug delivery.

## 5. Conclusions

Nanotechnology-based ocular delivery systems represent a rapidly evolving therapeutic approach for retinal diseases, such as age-related macular degeneration and diabetic retinopathy. The current findings summarized in this review highlight that by utilizing nanocarriers like liposomes and polymeric or lipid-based nanoparticles, researchers can significantly enhance drug stability, bioavailability, and targeted delivery, while navigating major anatomical and physiological barriers of the eye. A primary advantage of these systems is their ability for sustained, controlled drug release, offering a promising alternative to the invasive intravitreal injections currently considered the gold standard.

Evidence from preclinical models highlights the importance of optimizing physicochemical properties, including size, surface change, and encapsulation efficiency, in order to achieve effective drug delivery across ocular barriers. Nevertheless, long-term safety remains a critical concern, as nanoparticles may induce inflammation, oxidative stress or cytotoxic effects.

Consequently, while nanotechnology shows significant therapeutic potential over traditional methods, future standardized research is required to ensure safety and reproducibility of nanocarriers-based systems in treatment of posterior segment ocular diseases.

## Figures and Tables

**Table 1:** Glossary of key technical terms used in this manuscript.

Term	Definition
Zeta potential	The electric potential at the shear plane surrounding a nanoparticle in suspension, measured in millivolts (mV). It reflects the surface charge of the particle. Negative zeta potential (e.g., −20 to −40 mV) indicates a negatively charged surface, which can influence interactions with biological barriers and colloidal stability. Values beyond ±30 mV generally indicate good electrostatic stability.
Encapsulation efficiency (EE%)	The percentage of the total drug added during nanoparticle preparation that is successfully incorporated into the nanocarrier. High EE% (e.g., >70%) indicates efficient drug loading and is associated with prolonged sustained release and reduced drug waste.
Polydispersity index (PDI)	A dimensionless parameter (range 0–1) that describes the breadth of the particle size distribution in a nanoparticle suspension. PDI values below 0.2 indicate a narrow, homogeneous size distribution (monodisperse system), which is associated with greater formulation reproducibility and predictable biodistribution. Values above 0.5 indicate a broad, heterogeneous distribution.
Amphiphilic structure	A molecular architecture that contains both hydrophilic (water-attracting) and hydrophobic (water-repelling) regions within the same molecule. In nanocarriers such as liposomes or micelles, amphiphilic molecules (e.g., phospholipids) self-assemble in aqueous environments to form structures capable of encapsulating both water-soluble and lipid-soluble drugs simultaneously.
Mucoadhesion	The ability of a nanocarrier to adhere to mucosal surfaces (e.g., the conjunctival mucosa of the eye) through non-covalent interactions including hydrogen bonding, electrostatic forces, and van der Waals interactions. Mucoadhesive nanocarriers exhibit prolonged residence time on the ocular surface, enhancing drug absorption and bioavailability following topical administration.
Biodegradability	The capacity of a nanocarrier material to be broken down by biological processes (enzymatic hydrolysis, oxidation) into non-toxic metabolites that can be eliminated by normal physiological pathways. Biodegradable polymers such as PLGA are hydrolyzed to lactic acid and glycolic acid, which enter the Krebs cycle and are excreted as CO_2_ and water.
Receptor-mediated endocytosis	A cellular uptake mechanism in which surface ligands on a nanocarrier bind to specific receptors on the target cell membrane, triggering internalization of the nanocarrier–receptor complex via vesicle formation. This mechanism underlies the enhanced cellular uptake of targeted nanocarriers (e.g., cRGD-functionalized PLGA nanoparticles) by cells overexpressing the corresponding receptor (e.g., αVβ3 integrin in RPE cells under pathological conditions).

**Table 2:** Summary of ocular nanocarriers in selected research models.

Author (Year)	Disease	Type of Nanoparticle	Drug	Study Model	Main Findings
Amato et al. (2023) [21]	Diabetic retinopathy	Liposomes	Litosan G	In vivo (STZ-induced diabetic mice)	LipoLG reduced caspase-3; prevention of VEGF upregulation; improved bioavailability and efficacy
Liu et al. (2020) [25]	Age-related macular degeneration	Polymeric nanoparticles (PLGA, cRGD-functionalized)	Bevacizumab + dexamethasone	In vitro (ARPE-19 cells)	Inhibited angiogenesis and migration; induced apoptosis; good targeting and biocompatibility
Qiu et al. (2019) [26]	Diabetic retinopathy	Polymeric nanoparticles (PLGA)	Fenofibrate	In vivo (STZ-induced diabetic rats)	Sustained release (8 weeks); reduced vascular leakage, retinal edema, leukostasis, VEGF and ICAM-1levels
Beirampour et al. (2024) [37]	Ocular inflammation	Polymeric nanoparticles (PLGA)	Baricitinib	Ex vivo (corneas from the remains of pigs); in vitro	High EE%, suitable size maintaining stability, enhanced permeation

## Data Availability

No new data were created or analyzed in this study. Data sharing is not applicable to this article.

## Abbreviations

The following abbreviations are used in this manuscript:

AMD	Age-related macular degeneration
DR	Diabetic retinopathy
IVT	Intravitreal injections
BRB	Blood–retinal barrier
oBRB	Outer blood–retinal barrier
RPE	Retinal pigment epithelium
TJ	Tight junctions
PLGA	Poly(lactic-co-glycolic) acid
VEGF	Vascular endothelial growth factor
cRGD	Cyclic Arg-Gly-Asp
ARPE	Arising retinal pigment epithelia
ICAM-1	Intercellular Adhesion Molecule 1
LNPs	Lipid nanoparticles
SLNs	Solid lipid nanoparticles
NLCs	Nanostructured lipid carriers
AuNPs	gold nanoparticles
AgNPs	silver nanoparticles
PDI	Polydispersity index
EE%	Encapsulation efficiency
mV	millivolts
